# Symptoms, but Not a Biomarker Response to Inhaled Corticosteroids, Predict Asthma in Preschool Children with Recurrent Wheeze

**DOI:** 10.1155/2012/162571

**Published:** 2012-12-05

**Authors:** E. M. M. Klaassen, K. D. G. van de Kant, Q. Jöbsis, S. T. P. Høvig, C. P. van Schayck, G. T. Rijkers, E. Dompeling

**Affiliations:** ^1^Department of Paediatric Pulmonology, School for Public Health and Primary Care (CAPHRI), Maastricht University Medical Centre (MUMC), P.O. Box 5800, 6202 AZ Maastricht, The Netherlands; ^2^Department of General Practice, CAPHRI, MUMC, P.O. Box 5800, 6202 AZ Maastricht, The Netherlands; ^3^Department of Medical Microbiology and Immunology, Sint Antonius Hospital, P.O. Box 2500, 3430 EM Nieuwegein, The Netherlands; ^4^Department of Sciences, Roosevelt Academy, P.O. Box 94, 4330 AB Middelburg, The Netherlands

## Abstract

*Background*. A reliable asthma diagnosis is challenging in preschool wheezing children. As inhaled corticosteroids (ICS) are more effective in asthmatics than in children with transient wheeze, an ICS response might be helpful in early asthma diagnosis. *Methods*. 175 children (aged two–four years) with recurrent wheeze received 200 **μ**g Beclomethasone extra-fine daily for eight weeks. Changes in Exhaled Breath Condensate (EBC) biomarkers (pH, interleukin (IL)-1**α**, IL-2, IL-4, IL-5, IL-10, IFN-**γ**, sICAM, and CCL-11), Fractional exhaled Nitric Oxide (FeNO), airway resistance, and symptoms were assessed. At six years of age a child was diagnosed as transient wheezer or asthmatic. Adjusted logistic regression analysis was performed with multiple testing correction. *Results*. 106 transient wheezers and 64 asthmatics were analysed at six years of age. Neither changes in EBC biomarkers, nor FeNO, airway resistance, or symptoms during ICS trial at preschool age were related to asthma diagnosis at six years of age. However, asthmatics had more airway symptoms before the start of the ICS trial than transient wheezers (*P* < 0.01). *Discussion*. Although symptom score in preschool wheezing children at baseline was associated with asthma at six years of age, EBC biomarkers, airway resistance, or symptom response to ICS at preschool age could not predict asthma diagnosis at six years of age.

## 1. Introduction

Asthma is one of the most commonly occurring chronic diseases in children. It mostly develops in early childhood and is characterized by recurrent episodes of wheezing, breathlessness, and coughing due to airway inflammation and airway hyperresponsiveness [1]. However, in the majority of children symptoms are virally induced and transient (so-called transient wheezers) [2]. So, the associated airway symptoms are subjective and not specific for asthma. Moreover, young children are not able to perform lung function tests. Consequently, a diagnosis of asthma at young age has proven to be difficult [1, 2]. Objective measurements of airway inflammation require invasive procedures such as bronchoalveolar lavage, bronchial biopsy, and induced sputum and therefore are not routinely used. Effective asthma treatment is available in the form of inhaled corticosteroids (ICS). ICS are less effective in children with airway symptoms not due to asthma [1, 3]. As a result of difficulties in proper asthma diagnosis, true asthmatics are often “undertreated,” whereas children with transient symptoms are often “overtreated” [4]. Although international guidelines advocate a trial treatment with ICS for a likely diagnosis of asthma in young children, so far studies addressing response to ICS as a diagnostic tool in children have been scarce [1]. 

Exhaled Breath Condensate (EBC) is a noninvasive tool to assess biomarkers of airway inflammation [5]. Based on the ICS response of inflammation biomarkers in EBC at preschool age, a prediction of asthma at a later age might be possible. Besides, ICS-induced changes in Fractional exhaled Nitric Oxide (FeNO), airway resistance, and symptoms might contribute to an early asthma diagnosis. Therefore, our study aim was to assess whether an ICS response based on changes in biomarkers in EBC, FeNO, airway resistance, and symptoms in preschool-aged wheezing children has a predictive value for an asthma diagnosis at age six years. 

## 2. Methods

### 2.1. Study Population

Participants from the ADEM study (Asthma DEtection and Monitoring study registered at clinicaltrial.gov: NCT 00422747) were included. The ADEM study is a long-term case-control study that started in 2006 in The Netherlands and aimed to develop a noninvasive instrument for early asthma diagnosis by using biomarkers of airway inflammation in exhaled breath (condensate), and early lung function measurements (airway resistance). The study protocol has been described previously [6]. In total, 252 children aged two to four years were included: 202 children with recurrent wheezing symptoms (≥2 wheezing episodes until inclusion according to the International Study of Asthma and Allergies in Childhood (ISAAC) questionnaire) and 50 healthy controls without wheezing episodes until inclusion [7]. For the present analysis only the children with recurrent wheezing symptoms were included. 

### 2.2. Study Design

The main study objective was to assess whether an ICS response in biomarkers in EBC at preschool age was able to predict an asthma diagnosis at six years of age. Besides, the diagnostic value of the ICS response in FeNO, airway resistance, and symptoms for the prediction of asthma at age six years was assessed. For this purpose, an ICS trial was started in preschool children with recurrent wheeze. Airway medication (such as ICS and *β*_2_-agonists) was temporarily stopped prior to measurement. Children who were not able to discontinue ICS from four weeks before the trial were excluded from analyses. In case of clear symptoms of an airway infection, the measurement was postponed for four weeks. During the initial visit EBC was collected, FeNO and airway resistance were assessed, and questionnaires were completed (for details see below). Subsequently, recurrent wheezing children received 100 *μ*g ICS (Beclomethasone) extra-fine two times a day via the AeroChamber (Trudell Medical International, ON, Canada) for eight weeks. Symptom relieve by using salbutamol (Airomir, Teva Pharma NL, Haarlem, The Netherlands) distributed by an AeroChamber was allowed during the trial. At the end of the ICS trial, all measurements were reassessed. Compliance to ICS was assessed by weighting ICS canisters before and after the trial. Children who used a minimum of 80% of the prescribed medication were considered compliant (used for the per protocol subanalysis). Children were followed-up until six years of age. At this age a definite asthma diagnoses was made (see further). 

The study protocol was approved by the Dutch Central Committee on Research Involving Human Subjects (CCMO: NL17407.000.07/2007-001817-40, The Hague, The Netherlands). All parents gave written informed consent. 

### 2.3. Asthma Diagnosis

At the age of six years, a definite diagnosis (transient wheezer or true asthmatic) was made by two paediatric pulmonologists. An asthma diagnosis was based on symptoms, lung function (reversibility to a *β*_2_-agonist and bronchial hyperresponsiveness), and medication use. Next to this clinical assessment a diagnosis was assessed by a computer calculated algorithm. In this algorithm, asthma was diagnosed in case two out of the following three features were present: (1) positive symptoms (coughing ≥1 night(s) a week, ≥2 episodes of wheezing during the last twelve months, some days wheeze during colds or exercise or night time, two out of the following three items; some days wheeze during colds or during exercise in combination with night time coughing; every day, most days or several days wheeze during colds, during exercise or during the night time; cough during the night time) [7, 8], (2) presence of lung function abnormalities (a 20% fall in Forced expiratory volume in one second (FEV_1_) induced by a provocative concentration of histamine <2 mg/mL, or bronchodilator reversibility ≥9% (see below)) [9], and (3) ICS use. In case of disagreement between the clinical diagnosis and the computer calculated diagnosis, children were reassessed by the two paediatric pulmonologists who decided on the final diagnosis.

Bronchial hyperresponsiveness and reversibility measurements were performed according to the ERS guideline [10]. FEV_1_ was assessed in each child by means of the MasterScreen Pneumo (Jaeger, Wuerzburg, Germany). The highest FEV_1_ of three technically satisfactory curves was used for analysis. A challenge test was performed using histamine dissolved in physiologic saline in doubling concentrations from 0.032 to 16 mg/mL. Concentrations were increased until a fall of ≥20% (PC_20_) in FEV_1_ was measured or the final dilution was reached. Thereafter, all children directly received 400 *μ*g salbutamol, and FEV_1_ was reevaluated after fifteen minutes. The change in FEV_1_ was expressed as a percentage of the predicted value.

### 2.4. Biomarkers of Inflammation in Exhaled Breath Condensate

EBC was collected during ten minutes of tidal breathing into a mask with a two-way nonrebreathing valve connected to a closed glass condenser with recirculation unit developed at our institute [5]. Directly after EBC collection and pH measurement (Radiometer, Zoetermeer, The Netherlands), samples were stored at −80°C until further analysis. A selection of proinflammatory cytokines ((interleukin (IL)-1*α*), T-helper 1 cytokines (interferon-*γ* (IFN-*γ*) and IL-2), T-helper 2 cytokines (IL-4 and IL-5)), an antiinflammatory cytokine (IL-10), a chemokine (Eotaxin (CCL-11)), and an adhesion molecule (soluble Intercellular Adhesion Molecule 1 (sICAM1)) were used for analysis. These biomarkers were analysed by using the multiplex immunoassay (Luminex Corporation, Austin, TX, USA) as described previously [5, 11–13]. Values under the detection limit received a randomly generated value between zero and the detection limit [12].

### 2.5. Fractional Exhaled Nitric Oxide

As previously described, FeNO was collected offline in a 500 mL inert balloon during tidal breathing and measured by using a nitric oxide monitoring system (NIOX, Aerocrine, Solna, Sweden) [6].

### 2.6. Airway Resistance

Airway resistance was measured during tidal breathing by means of the MicroRint (Micro Medical, Rochester Ltd, UK) as described before [6, 14]. The median of at least 5 technically acceptable airway interruptions during an expiratory peak flow was used for analysis. Measurements were performed before and fifteen minutes after inhalation of 300 *μ*g of salbutamol via a spacer (AeroChamber). 

### 2.7. Symptom Score

Parents completed a questionnaire on airway symptoms [8]. Based on this questionnaire a symptom score was computed by adding six items (cough during daytime, cough at night, wheeze during daytime, wheeze at night, dyspnoea during daytime, and dyspnoea at night) on a Likert scale (every day = 1, most days = 2, some days = 3, a few days = 4, and not at all = 5). The minimal score was six, and the maximal score was 30 (no day and night time symptoms).

### 2.8. Statistical Analysis

Data were analysed using SPSS 18 (SPSS Inc., Chicago, IL, USA). Due to the skewed distributions, biomarkers in EBC were log transformed which successfully imparted a normal distribution. Differences in baseline characteristics between transient wheezers and true asthmatics were evaluated by using the chi-square test for categorical variables and the independent *t*-test for continuous parametric variables. The effect of each individual potential predictor (both before the ICS trial and changes during ICS) on asthma diagnosis at six years of age was tested by separate logistic regression models. Models were adjusted for possible confounders (gender, atopy, eczema, previous ICS use, first degree relative with asthma, smoking exposure, and season of measurement). Data were presented for intention to treat analysis unless otherwise specified. *P* values were corrected for multiple testing by False Discovery Rate (FDR) for each hypothesis [15]. Differences were considered statistically significant when the FDR corrected *P* value was <0.05.

### 2.9. Power Analysis

The primary outcome measure is the definite asthma diagnosis at six years of age. In a population of young children with recurrent airway symptoms, the prevalence of asthma at six years of age is 30% [16]. In the current study, including 202 children with recurrent wheeze, this will result in at least 50 children with asthma taken into account a drop out rate of 10%. Based on an earlier study in children with asthma and healthy controls aged 6–16 years, differences in concentrations of IL-1*α*, IL-2, IL-4, IFN-*γ*, CCL-11, and sICAM were found of, respectively, 4.5, 13.2, 2.9, 11.3, 0.9, and 80.0 pg/mL [17]. If we assume comparable differences in for example CCL-11 in the present population between asthmatics and transient wheezers with a power of 90% and an alpha of 0.05, 39 asthmatics and 78 transient wheezers are needed. 

## 3. Results

### 3.1. Baseline Characteristics

**Figure 1:**
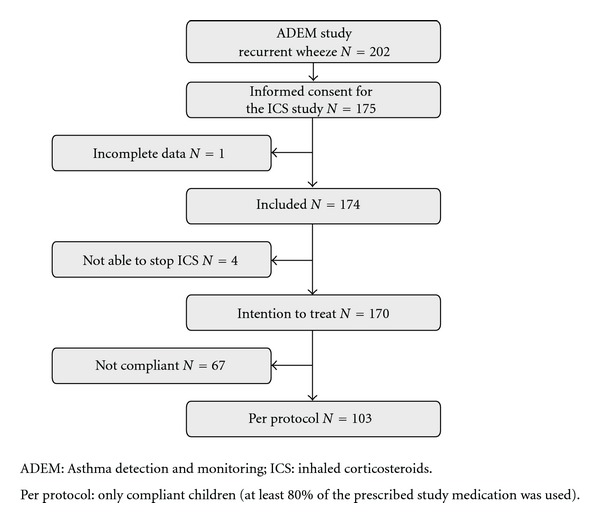
Study flow chart.

Of the 202 recurrent wheezers included in the ADEM study, 175 parents gave informed consent to participate in the ICS trial. One participant was excluded from analysis as an asthma diagnosis could not be assessed due to missing data. Four children (all true asthmatics) were unable to stop their regular ICS medication and were therefore excluded from further analysis. In total, 170 (65 asthmatics and 105 transient wheezers) children were included in the intention to treat analysis and 103 (42 asthmatics and 61 transient wheezers) in the per protocol analysis (as they used a minimum of 80% prescribed ICS).  Figure 1 presents the study flow chart. Agreement between clinical and computed diagnosis was reached in 83% before and 89% after clinical reassessment. 

Baseline characteristics are displayed in Table 1. Preschool children in the asthmatic group used significantly more ICS and short-acting *β*_2_-agonists in the preceding year compared to the transient wheeze group. Besides, atopy at preschool age was significantly more frequent in true asthmatics. At six years of age, ICS and short-acting *β*_2_-agonists were used in 40/43% of the asthmatics and 2/6% of the transient wheezers, respectively. Atopy was present in 45% of the asthmatics and 31% of the transient wheezers at six years of age.

### 3.2. Analysis at Baseline

EBC and FeNO were successfully collected in all children (100%). With the exception of Eotaxin (89%), all EBC markers were above the detection limit in more than 98% of the samples. Prebronchodilator and postbronchodilator airway resistance were successfully assessed in 95% and 97% of the children, respectively. The symptom score was present in all children (100%). No statistically significant differences in biomarkers of inflammation in EBC, FeNO, or airway resistance were observed between both groups at preschool age (Table 2). However, children with an asthma diagnosis at six years of age had significantly more symptoms (lower symptom score) at preschool age compared to transient wheezers (odds ratio = 0.85; 95% confidence interval = 0.77–0.93; *P* < 0.01 FDR corrected).

### 3.3. Changes Induced by Inhaled Corticosteroids

No statistically significant changes between transient wheezers and asthmatics in biomarkers in EBC, FeNO, airway resistance or symptom score were induced by the ICS trial (Table 3). FeNO levels in the total group neither showed significant changes (median ppb (interquartile range) before the ICS trial: 8.4 (4.2–14.3), after the ICS trial: 9.6 (4.5–15.4); *P* = 0.45).

### 3.4. Subanalysis per Protocol

Results of the study did not change when only compliant children were included in the analysis (per protocol analysis).

## 4. Discussion 

Presently, no adequate diagnostic tool is available for an early asthma diagnosis in preschool wheezing children. This study addressed the question whether response to ICS treatment of biomarkers of inflammation in EBC, FeNO, airway resistance, and symptoms at preschool age could predict an asthma diagnosis at six years of age. Changes in biomarkers in EBC, FeNO, airway resistance, or symptoms induced by an eight-week ICS treatment at preschool age were not related to an asthma diagnosis at six years of age. However, it was demonstrated that children with asthma at six years of age had more respiratory symptoms at preschool age compared to transient wheezers. Also, medication use and atopy at baseline were significantly higher in asthmatics compared to transient wheezers. 

Collection of EBC is a noninvasive method in which biomarkers expected to reflect airway inflammation can be assessed, and most influence of the systemic metabolism is avoided. Previously our research group has shown differences of biomarkers of inflammation between asthmatics and healthy children [17]. Moreover, a higher concentration for multiple biomarkers was noted in children with more severe wheeze compared to children without wheeze in the present population [13]. In adult populations with asthma, it was demonstrated that several biomarkers in EBC significantly changed after ICS treatment [18, 19]. Differences of ICS response based on changes in biomarkers in children have not yet been investigated. In the present study, none of the selected biomarkers in EBC before or changes during the ICS treatment were associated with an asthma diagnosis at six years of age. Besides, FeNO assessed before or a change during ICS treatment at preschool age was not associated with an asthma diagnosis at six years of age. FeNO levels in the total group neither showed significant changes. Data on the effectiveness of predicting an ICS response by FeNO in children are conflicting [20–22]. Asthma is expected to be predominated by an eosinophilic airway inflammation while transient wheeze is characterised by a neutrophilic inflammation [23–25]. ICS are believed to mainly have an antieosinophilic inflammation effect [1, 3, 24]. Therefore, ICS are expected to be more effective in asthmatics compared to transient wheezers. However, increasing evidence points towards overlap between eosinophilic and neutrophilic inflammation in both asthma and transient wheeze [26, 27]. Due to this overlap, changes in inflammation biomarkers induced by ICS treatment might be less pronounced, and these changes may not be able to distinguish between transient wheezers and true asthmatics, as was observed in our study. It would be interesting to study differences with healthy children as well. Previously we did demonstrate differences between baseline measurements of biomarkers in EBC in healthy children and children with recurrent wheeze in the same cohort [12]. However, due to ethical constraints the healthy children have not been included in the trial.

Airway resistance in preschool children has been found to be predictive for asthma symptoms at six years of age [28]. Besides, bronchodilator reversibility was able to predict effectiveness of anti-inflammatory therapy in young children with asthma [20]. No ICS response for airway resistance or reversibility was demonstrated in the present study. These results should be interpreted with caution as the children participating in our study in general had mild symptoms before and during ICS treatment, thus leaving little room for improvement. 

Even though most children had only mild symptoms, symptoms at preschool age were positively associated with an asthma diagnosis at six years of age. This association remained highly significant after multiple testing corrections. However, changes in symptoms during ICS treatment were not related to the asthma diagnosis at six years of age. This result can be interpreted twofold. First, as symptoms were mild there was not enough room for improvement during ICS treatment. Second, improvement in symptoms was equal for asthmatics and for transient wheezers. Either way, differentiation of true asthmatics from transient wheezers based on symptom reduction due to ICS treatment was not possible in our study. It will be interesting to address this issue in a population with more severe symptoms.

The major strength of this study is the longitudinal design that enabled the assessment of an ICS response of several biomarkers at preschool age and an asthma diagnosis at six years of age. A second strength of this study is the high follow-up rate. Information to assess an asthma diagnosis was available in all but one child at six years of age. Thirdly, measurements were feasible and noninvasive which made them suitable for young children. Feasibility of assessing biomarkers in EBC was increased by the use of a double-jacketed condenser with heated breath recirculation unit as was developed at our institute [5]. Due to this efficient condenser system EBC could be collected in a relatively short collection time. Besides, multiple biomarkers could be measured in a small sample by the highly sensitive multiplexed liquid bead arrays with good to excellent reproducibility [11, 17]. Finally, we chose to administer Beclomethasone extra-fine. Previous research demonstrated that the use of small particles in young children are effective and safe and are expected to result in increased and more uniform deposition in the lower respiratory tracts. This minimizes the necessary dosage for administration [29, 30]. However, some limitations need to be addressed. Firstly, measurement of biomarkers of inflammation in EBC is a technique that still needs more validation and standardisation. Despite these restrictions, our research group has extensive experience on the use of EBC as a noninvasive technique to assess biomarkers in airway inflammation [5, 6, 17]. Secondly, as asthma is heterogeneous by nature, difficulties might arise when assessing an asthma diagnosis. In the present study, both a clinical and a computer algorithm were used for an asthma diagnosis. The degree of accordance between the clinical and algorithm diagnosis was high, and mismatches were reevaluated in order to prevent an incorrect diagnoses. Finally, it can be argued whether a period of eight weeks ICS is sufficient to assess changes in biomarkers in EBC, FeNO, airway resistance, and symptoms at preschool age to predict an asthma diagnosis at six years of age. According to international guidelines a treatment trial with ICS of at least eight weeks in preschool children can give guidance to the presence of asthma [1]. Therefore, changes in biomarkers in EBC, airway resistance, FeNO, and symptoms are to be expected after eight weeks.

## 5. Conclusions

In conclusion, in this study we demonstrated that, although symptoms at preschool age were predictive for an asthma diagnosis at age six years, changes in biomarkers of inflammation in EBC, FeNO, airway resistance, and symptoms induced by ICS at preschool age could not predict an asthma diagnosis at six years of age. In the absence of a proper diagnostic tool for asthma at young age, adequate care and management of children with airway symptoms remains hampered. Although no predictive value of biomarkers in EBC could be demonstrated in the present study, EBC biomarkers might still be of additional value for an asthma diagnosis. In the future, we will determine whether profiles of EBC biomarkers in combination with other parameters such as clinical features, exhaled volatile organic compounds, and gene expression can be of value in predicting an asthma diagnosis.

## Figures and Tables

**Table 1:** Characteristics at baseline for transient wheezers and true asthmatics.

	Transient wheeze *N* = 105	Asthma *N* = 65	Total *N* = 170
Mean age (SD)	3.3 (0.6)	3.3 (0.6)	3.3 (0.6)
Gender: male/female (*n*)	55/50	37/28	92/78
Atopy^†^, %*	20	35	26
Eczema, %	36	47	40
Use of ICS, %*	12	26	18
Use of short-acting *β*_2_-agonists, %*	31	54	40
Asthma first degree relatives, %	36	44	39
Smoking exposure, %	30	31	31

*P < 0.05 between transient wheezers and true asthmatics; ^†^atopy is defined as a positive (≥0.35 kU/L); Phadiatop Infant test (Phadiatop Infant, Phadia, Uppsala, Sweden); SD: standard deviation.

**Table 2:** Baseline analysis at preschool age.

	Median (IQR) transient wheeze	Median (IQR) asthma	OR	95% CI
IL-1*α*, in pg/mL	34.0 (15.6–83.7)	44.2 (18.3–97.6)	1.10	0.84–1.44
IL-2, in pg/mL	55.5 (35.7–76.8)	57.0 (43.1–81.2)	1.32	0.80–2.16
IL-4, in pg/mL	8.8 (4.8–13.9)	9.6 (6.0–14.5)	1.15	0.70–1.88
IL-5, in pg/mL	29.8 (17.8–58.7)	37.2 (19.4–80.6)	1.21	0.85–1.73
IL-10, in pg/mL	4.5 (2.8–6.9)	5.2 (3.2–8.4)	1.09	0.72–1.65
IFN-*γ*, in pg/mL	28.1 (17.5–44.6)	27.1 (16.0–44.2)	0.88	0.67–1.16
sICAM, in pg/mL	190.4 (94.8–416.2)	265.5 (104.1–516.4)	1.08	0.79–1.48
CCL-11, in pg/mL	8.3 (5.2–12.5))	8.0 (5.3–14.6)	0.90	0.71–1.15
pH	6.0 (5.7–6.3)	5.8 (5.6–6.2)	0.66	0.34–1.28
FeNO, in ppb	6.7 (3.7–13.3)	8.9 (5.4–18.1)	1.02	0.98–1.05
Airway resistance before medication^§^	1.4 (0.3)	1.5 (0.4)	2.15	0.83–5.56
Airway resistance after medication^§^	1.2 (0.4)	1.3 (0.3)	2.06	0.70–6.01
Bronchodilator response^§^	0.1 (0.3)	0.0 (0.5)	1.44	0.45–4.55
Total symptom score*	27 (25–29)	25 (20–28)	0.85	0.77–0.93

*False Discovery Rate (FDR) corrected *P* < 0.01; ^§^mean (standard deviation).

IL: interleukin; IFN-*γ*: interferon-*γ*; sICAM: soluble Intercellular Adhesion Molecule; CCL-11: Eotaxin; FeNO: Fractional exhaled Nitric Oxide; OR: odds ratio; 95% CI: 95% confidence interval. Analysis adjusted for gender atopy, eczema, previous inhaled corticosteroids use, asthma first relative, smoking exposure, and season of measurement. The transient wheeze group is the reference group.

**Table 3:** Changes induced by inhaled corticosteroids related to an asthma diagnosis.

	OR	95% CI
IL-1*α*	0.96	0.78–1.19
IL-2	0.99	0.71–1.38
IL-4	0.90	0.64–1.28
IL-5	0.97	0.76–1.24
IL-10	0.87	0.65–1.16
IFN-*γ*	0.93	0.73–1.20
sICAM	0.97	0.77–1.22
CCL-11	0.94	0.75–1.17
pH	0.99	0.61–1.60
FeNO	1.01	0.99–1.04
Airway resistance before medication	0.80	0.33–1.93
Airway resistance after medication	1.51	0.55–4.16
Total symptom score decreased	Ref.	
Total symptom score equal	0.74	0.27–2.04
Total symptom score increased	0.65	0.30–1.39

IL: interleukin; IFN-*γ*: interferon-*γ*; sICAM: soluble Intercellular Adhesion Molecule; CCL-11: Eotaxin; FeNO: Fractional exhaled Nitric Oxide; OR: odds ratio; 95% CI: 95% confidence interval; Ref.: reference category. Analysis adjusted for gender atopy, eczema, previous inhaled corticosteroids use, asthma first relative, smoking exposure, and season of measurement. The transient wheeze group is the reference group. Due to violation of linearity assumption in log transformation, results for total symptom score are split up into three equal categories.
